# What about Happiness? A Critical Narrative Review with Implications for Medical Education

**DOI:** 10.5334/pme.856

**Published:** 2023-06-06

**Authors:** Fabienne Schwitz, Jacqueline Torti, Lorelei Lingard

**Affiliations:** 1 Cardiologist and medical educator, Department of Medicine, Schulich School of Medicine and Dentistry, Western University, Medical Sciences Building, Suit 102A, London, Canada; 2 Department of Cardiology, Inselspital Bern University Hospital, University of Bern, CH 3010 Bern, Switzerland; 3 Department of Medicine, Schulich School of Medicine and Dentistry, Western University, Medical Sciences Building, Suit 102A, London, Canada

## Abstract

**Introduction:**

Despite abundant scholarship and improvement initiatives, the problem of physician wellbeing persists. One reason might be conceptual: the idea of ‘happiness’ is rare in this work. To explore how it might influence the conversation about physician wellbeing in medical education, we conducted a critical narrative review asking: ‘How does happiness feature in the medical education literature on physician wellbeing at work?’ and ‘How is happiness conceptualized outside medicine?’

**Methods:**

Following current methodological standards for critical narrative review as well as the Scale for the Assessment of Narrative Review Articles, we conducted a structured search in health research, humanities and social sciences, a grey literature search, and consultation with experts. After screening and selection, content analysis was performed.

**Results:**

Of 401 identified records, 23 were included. Concepts of happiness from the fields of psychology (flow, synthetic happiness, mindfulness, flourishing), organizational behaviour (job satisfaction, happy-productive worker thesis, engagement), economics (happiness industry, status treadmill), and sociology (contentment, tyranny of positivity, coercive happiness) were identified. The medical education records exclusively drew on psychological concepts of happiness.

**Discussion and Conclusion:**

This critical narrative review introduces a variety of conceptualizations of happiness from diverse disciplinary origins. Only four medical education papers were identified, all drawing from positive psychology which orients us to treat happiness as individual, objective, and necessarily good. This may constrain both our understanding of the problem of physician wellbeing and our imagined solutions. Organizational, economical and sociological conceptualizations of happiness can usefully expand the conversation about physician wellbeing at work.

## Introduction

For more than 30 years, we have been discussing the issue of physician wellbeing at work. Scholarship on physician wellbeing and related concepts such as life satisfaction and wellness has: analysed contributors and impacts of burnout [1,2,3], identified health problems among physicians and potential solutions [1,4,5], explored wellness and the pursuit of balance [6,7,8], and implemented interventions to improve resilience among physicians [9,10,11,12]. This scholarship has informed a movement to improve wellbeing in medical education and medicine [13,14]. Main dimensions of this movement are: changing of organizational strategies [1,12], promoting the wellbeing of physicians in training through faculty development [13,15] and systematic strengthening of individual factors [1,12,15].

Despite this wealth of scholarship and improvement initiatives, the problem of physician wellbeing seems to have improved very little [15]. Data from the Association of American Medical Colleges Graduation Questionnaire and Year 2 Questionnaire from 2016 to 2019 show no sign of improvements among medical students despite targeted efforts [16,17]. The Accreditation Council for Graduate Medical Education also surveys residents and faculty annually, but changes to wellbeing in recent years are challenging to analyze due to a change in the structure of the questionnaire and the onset of the pandemic. What is clear is that mental illness and suicide among doctors has increased [18], supported by data highlighting high rates of mental health symptoms among physicians [19]. Furthermore, the COVID-19 pandemic appears to have worsened the situation. Its potential impact on the mental wellbeing of health workers has been studied [20], with consistent reports of stress, anxiety and depressive symptoms among healthcare professionals [21]. Thus, the question of why physician wellbeing efforts are unsuccessful is particularly pressing.

One reason might be conceptual. Amid the suite of concepts around which physician wellbeing work has formed, the notion of ‘happiness’ does not strongly feature. Happiness is a richly theorized construct in other domains. It is used for a number of different constructs [22,23], and a distinction between affective wellbeing and eudaimonia or eudaimonic wellbeing (meaning and purpose of life) has been recognised since antiquity [22]. While the two conditions, wellbeing and happiness, are related (or even perhaps conflated) in the literature, their relationship is not clear. This review arises from our assumption that the term ‘happiness’ may carry additional, distinctive meanings that could influence the conversation about physician wellbeing in medical education. Other scholars have had similar ideas: e.g., Alan Peterkin has noted that ‘pleasure and happiness are neglected in our training and in our work’ [24]. Thus, we conducted a critical narrative review guided by two questions: ‘How does happiness feature in the medical education literature on physician wellbeing?’ and ‘How is happiness conceptualized outside medicine?’ We aim to describe whether and how happiness features in medical education, how the construct of happiness is understood in other select domains, and how we might adopt understandings from other domains to enrich the scholarship of physician wellbeing at work going forward.

## Methods

This critical narrative review followed the Scale for the Assessment of Narrative Review Articles (SANRA) procedure [25] and was also informed by Kahlke’s work [26]. Our goal has been to explore a wide-ranging literature and to be deliberately selective in highlighting works that allow us to achieve the key contribution of a narrative review: deepened understanding that advances the scholarly conversation [27].

Our review began with the question ‘How does happiness feature in the medical education literature on physician wellbeing?’ ‘Happiness’ is a broad and multifaceted concept, one which a single review would struggle to comprehensively capture; given our interest in physician happiness at work, we used the term “workplace happiness” to focus our inquiry. We conducted a structured literature search in the medical literature for ‘workplace happiness AND physicians’ in the databases PubMed, CINHAL and PsycINFO with librarian assistance. In the screening process, we excluded records that contained the term ‘happiness’ (or derivatives such as ‘happy’) in passing, without definition or conceptualization. For example, one article mentioned that 52% of pathologists are ‘happy’ with their job but did not describe how happiness was understood [28].

Having identified the records in medical education, our next step was a humanities and social sciences literature search to address the second question, ‘How is the term happiness used outside medicine?’ We searched using purposeful combinations of ‘workplace happiness’ and ‘happiness’ in Web of Science, Embase and Scopus. Searches in the grey literature covered a broad spectrum from Google Scholar to TED Talks. Discussion with experts from psychology, sociology, philosophy, women studies and medical education helped us identify relevant sources. We did not use synonyms for happiness in our search, as our intent was not a full exploration of the conceptualization of happiness but rather an understanding of how these disciplines use the term happiness.

The authors judged the sufficiency of our database on two levels. First, we sought to represent a range of disciplines in which happiness is theorized. Because our structured search returned sources from sociology, psychology, economics and organizational behaviour, these became the focus of our results. Critical narrative review involves compiling a selective rather than comprehensive set of records; we judged this range of disciplines sufficient to provide useful insights on which to base future research. The second point of sufficiency relates to how many and which records from each discipline are necessary to represent key notions around happiness. Rather than setting a threshold for a specific number of records, the authors instead considered whether the records offered a sufficiently robust description of the concept. As we analysed selected records, we judged them sufficient once we could explain the concept and recognize redundancies in additional records. Where we could not make this judgment, we sought additional records. Overall, we justified the choice of literature based on its ability to support a description of different conceptualizations of happiness that could advance our thinking on the topic in medical education. This process is the essence of rigour in critical narrative reviews [26].

Screening and selection were shaped by the orientations of our research team members. Our group includes a senior medical education researcher with disciplinary roots in the humanities, an early career medical education researcher with roots in public health and health promotion, and a cardiology educator completing a masters in medical education. We brought to the review different degrees of familiarity and comfort with the disciplines we were searching: for instance, we were all familiar with medical education scholarship, but two of us were also familiar with scholarship and vocabulary from social sciences, psychology and organizational behaviour, while none of us were familiar with economics scholarship. This invariably influenced our insights and enthusiasms for particular concepts retrieved in our search, but together we endeavoured to address all retrieved records with the same attention and support each other to interpret those we were less familiar with.

After screening and selection of records for both the focused medical education search and the broader, multi-discipline search, content analysis was done to enhance the trustworthiness of the study. Content analysis offered a way to arrange the data systematically into distinct categories, including definitions and conceptualizations of happiness, disciplinary origin of ideas, connections and similarities, and differences or tensions. Concept mapping enabled us to visualize the relationships among these categories, and informed our interpretation of these relationships.

## Results

**Figure 1:**
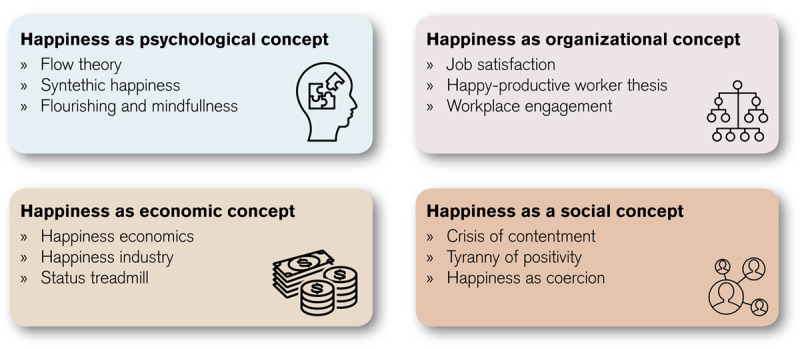
Disciplinary patterns in the characterization of happiness.

Our searches retrieved 401 records. After abstract and title screening, 28 were selected for full-text review, of which 13 were included. An additional 10 were included from hand-searching and grey literature for a total of 23. Our focused medical education search yielded 4 records. Our second, broader search yielded records from psychology (8), organizational behavior (6), economics (2) and sociology (3). While such disciplinary categories are necessarily simplifications, we use them in the following sections as organizing structures to highlight disciplinary patterns in how the records characterize happiness (Figure 1: Disciplinary patterns in the characterization of happiness). Because our analysis of the medical education records showed that this scholarship exclusively employed psychological concepts, we have included these records (which address our first research question) in that section. Because of the small number of medical education records, the bulk of our results address our second research question. Terminologies vary and are used interchangeably in this literature (Cromby cited in Frawley) [29]; however, for consistency we deliberately use the term ‘happiness’ except when quoting from a record that uses another term.

### Happiness as a psychological concept

Psychological concepts of happiness have in common a predominantly individual approach. In this section, we highlight four psychological concepts that have particular relevance in the context of physician wellbeing at work, including those that have already been taken up in medical education.

The first concept is flow theory [30], which states that you need a flow feeling – a state of ideal balance between challenges and abilities—to be happy. Flow is a state of being focused; having a sense of ecstasy, inner clarity and serenity; knowing that an activity is doable; feeling timelessness and intrinsic motivation [31]. Flow tends to occur at work more than in leisure time [32]: e.g., a musician could reach a flow state during a concert. The concept of flow has its origin in positive psychology. The initial study about flow theory by psychologist Csíkszentmihályi included surgeons, and the concept of flow has been taken up in medical education in a paper about enhancing career enjoyment, performance and workplace happiness [33,34]. This work supported flow through training in individual interventions such as coaching, psychological skills training, mental skills training or stress management training [33]. While supporting flow in health care is recognized to require supplementary system-level interventions, such as increasing time for patient care, minimizing administrative tasks, and promoting constructive learning environments, scholarship at the system level is less common [33,35].

The second concept from positive psychology is synthetic happiness. The term was coined by Daniel Gilbert in a 2004 TED Talk, although his subsequent writings do not use it. Contrasted with ‘natural happiness’ which is what we feel when we get what we want, ‘synthetic happiness is what we make when we do not get what we want’ [36]. A term coined by Harvard psychologist Daniel Gilbert, synthetic happiness acts like a psychological immune system, to ‘[strike] a balance that allows us to feel good enough to cope with our situation but bad enough to do something about it’ [36,37]. We convince ourselves that we have is what we would have chosen anyway. For instance, clinical clerkship students acknowledge what they like or dislike about each specialty as they rotate through them, but during residency their choice is already made, so they are likely to convince themselves that they are happy with it. Synthetic happiness helps us to find a way to like what we have. However, synthetic is not ‘false’; Gilbert argues that ‘synthetic Happiness is as real and enduring as the natural happiness’ [36], suggesting it could have implications for being happy in an imperfect workplace.

Finally, two related concepts from positive psychology occurred in our records: flourishing and mindfulness [38,39]. Flourishing draws on the metaphor of flower growth, and refers to a lifestyle determined by optimal living, mastering life’s tasks, kindness, personal growth, and resilience. Five pillars are required: positive emotion, engagement, relationships, meaning, and accomplishment [40]. These factors form the basic precondition for a life of profound fulfilment. Mindfulness is described as a mental state achieved by focusing one’s awareness. While it shares features with flourishing, it is the practice of non-judgmental attention in the moment. Only one record used mindfulness as part of a suite of interventions to ‘increase happiness’^4^; the others did not articulate increased happiness as an explicit goal of mindfulness.

### Happiness as an organizational concept

Organizational approaches to happiness arise from the premise that individuals spend most of their adult lives working. As such, happiness in the workplace is a key component of overall happiness in life. In this section, we highlight key concepts in our records that were associated with happiness at work, including job satisfaction, productivity, and employee engagement.

A common idea in the organizational literature about happiness is ‘job satisfaction’. Components of job satisfaction that contribute to employee happiness include aspects at the transient, individual (employee) and organizational or collective level [41,42]. Transient-level contributions to workplace happiness include momentary affect, emotion at work and flow state [43]. Employee-level contributions to workplace happiness include job security, meaningful work, positive relationships with coworkers, recognition, autonomy and engagement [44]. The organizational aspects that contribute to workplace happiness include opportunities for growth, compensation, job flexibility, a positive work environment, work-life balance, and organizational culture [44]. Both the employer (leader) and the individual (follower) play a strong role in shaping organizational aspects that contribute to happiness through constructive dissensus [45]. Constructive dissensus refers to a mutual understanding between leaders and followers, including shared beliefs and values, leading to improved quality of life at work. Interventions aimed at improving workplace happiness should be multi-faceted and focus both on improving individual happiness as well as the organizational aspects to enhance job performance and employee retention [45].

The happy-productive worker thesis states that all things being equal, happy workers perform better than those who are less happy. This idea has been the holy grail of management and organizational psychology research for two decades. With roots in positive psychology, the happy-productive worker thesis explicitly focuses on ‘productivity as a consequence of happiness at work’ [46]. Happiness in this thesis was operationalized by a diversity of constructs (affect, wellbeing, burnout, life satisfaction, growth and purpose), which may explain the inconclusive nature of the empirical literature, which finds that people who are happy in general are more productive, but people who are happy specifically at work are not necessarily so.

‘Engagement’ was a crucial dimension of measuring happiness at work [47]. Workplace engagement is defined as a persistent affective state that includes how employees express themselves physically, cognitively, and emotionally at work. The physical aspect of engagement focuses on the energy expended by individuals to perform their job, while the cognitive aspect focuses on employees’ beliefs about their organization and the emotional aspect focuses on how employees feel towards the organization [48]. Workplace happiness is the ‘positive outcomes at the workplace which are a result of many interlinked factors ranging from employee-work relation within the organization to the end results of efficient production and customer satisfaction’ [47]. The organizational behavior literature connects engagement to productivity. Describing engagement as having a positive correlation with constructs like organization growth, lower operational costs, lower absenteeism, and decreased intentions to turnover [47], the organizational literature begins to articulate an economics of happiness which is even more explicit in the economic records we analyzed.

### Happiness as an economic concept

Economic approaches to happiness arise from the position that happy workers improve the economy. Therefore, the search for happiness has also become more and more of a business. In this section, we highlight the aspects related to the ‘happiness industry’.

Happiness is a central concept in economics. Global institutions such as the World Bank and the World Health Organization embrace the relationship between subjective happiness and economic wellbeing [29], and, since 2012, the World Happiness Report has tracked the happiness of countries as a marker of economic health. ‘Happiness economics’ conceives of happiness as an objective, measurable entity, something that can be ‘reduced to calculable units’ [29]. Happiness is calculated at individual and group levels, with the goal of converting happiness into familiar kinds of economic capital like gross domestic product, consumer spending, and employment rates.

With the rise of the happiness industry, the interaction between happiness and the markets becomes a central concern of economics. As a marketing device, happiness is strategic. Consumer groups must be poised ‘between pleasure and pain’: just happy enough to sustain the idea that products bring happiness and just unhappy enough to feel the need to buy more [49]. However, economic growth compulsion can have a negative impact on happiness, as consumers are confronted with the agony of choice. Multiple options can only contribute to happiness as long as the number of options is still manageable. Once a certain threshold is reached, additional options do not bring further happiness. And purchasing power is not the solution.

Studies of the relationship between income and happiness suggest that, despite economic growth and increased prosperity, people are not happier. At the same time, however, critical economic scholars warn against extrapolating that poorer means happier (Pender cited in Frawley) [29], and raise concern that the emphasis on the subjective sensations of the populace can deflect attention from objective realities like insufficient food. Furthermore, the relationship between money and happiness is complex: the ‘status treadmill’ concept captures the notion that it is relative, not absolute, income that can lead to happiness, and we are happier when our relative income is higher than our peers [50]. In relation to physician happiness at work, these economic concepts suggest a complex, nonlinear relation between physician income and happiness.

### Happiness as a social concept

Sociological approaches to happiness arise from the position that the predominantly psychological treatment of happiness fails to attend to the affordances and impediments to happiness that exist at a structural level. In this section, we highlight two concepts – the crisis of contentment and happiness as coercion – that arose in our records.

In the book, Deconstructing Happiness, McKenzie describes a crisis of contentment. They distinguish happiness, which is an individual characteristic, from contentment, which is ‘a collective social project’ [51] arising from ‘committing to something greater than the self’ [51]. Contentment is socially defined and motivated, standing in contrast to pleasure-driven, temporary and individual forms of happiness. With this distinction, the author argues that we are currently experiencing a ‘crisis of contentment’ (not of happiness) in modern society. Our lives are ‘filled with an almost unending range of products, services and self-help books that will lead to happiness’ but ‘people do not seem to be as happy as they SHOULD be’ [51] because individual happiness cannot provide meaning and long-term satisfaction without context. The values and norms of the social world provide this context. Therefore, ‘how the individual is able to positively place him-or herself with regard to social values and norms’ [51] is the basis of contentment.

The concept of a crisis of contentment moves us away from concerns about the individual’s happiness or lack thereof. Instead, we must be concerned with the social, political and economic factors that shape the relationship between the individual and society [51]. In particular, we need to critique our *expectations* of happiness [29]. Some sociologists have argued that the idea of happiness is part of a widespread ‘tyranny of positivity’ in modernity, pressuring individuals ‘to conform to culturally sanctioned ideals of behavior and disposition’ [51]. A growing body of work challenges the ‘epistemological fallacy that happiness ‘has’ a kind of essence’ and argues instead that the concept of happiness is a relational construct, gaining meaning from socio-historical context and attendant social norms’ [51]. Happiness, they argue, is not a neutral concept.

Happiness as coercion is an instance of its lack of neutrality, as argued by critical feminist scholarship. For instance, Sara Ahmed considers happiness as a ‘world-making device’ [52]: the world is made by prescribing what is appropriate ‘happiness’ based on largely unacknowledged social values and norms. Happiness, then, is an instrument for reorienting the individual toward a common good. Thus, as ‘an idea or aspiration within everyday life’ happiness can lead to ‘forms of coercion …such that one person’s happiness is …made conditional on their willingness to be made happy by the same things as other people’. Happiness, therefore, makes demands on us [50]. It demands that we accept, with a smile, the social values that have dictated what happiness is. Using the example of ‘happy housewife’ of the 1950s, she argues that this notion of female happiness enforces ‘gendered forms of labour’ and encourages us to consider the possibility of refusing to be happy in the ways that society prescribes. What would it mean instead to ‘claim the freedom to be unhappy’ [52]?

## Discussion

This critical narrative review has articulated multiple conceptualizations of happiness that could inform the way we approach physician wellbeing at work in medical education. In this section, we provide three main insights about these conceptualizations of happiness and consider their implications for scholarship and improvement efforts related to physician wellbeing in the workplace.

### Happiness is not only individual: it is also social

The four medical education papers we analysed that explored physician happiness at work all relied upon concepts of happiness based in positive psychology. And while efforts to promote physician flow and mindfulness at work are valuable, they represent a partial view of the phenomenon due to their focus on the individual. Even in the Canadian Medical Association Journal call to consider ‘Physician health: beyond wellness to happiness’, the emphasis is on personal attributes such as stoicism and perfectionism. However, as McKenzie argued, ‘the individual approach to happiness is both incomplete and inherently flawed’ [51]. We would argue that we’ll never fully understand physician happiness by exclusively emphasizing individual psychology. We need to also address the relationship between the individual and society by considering the concept of contentment, which requires paying attention to social, political and economic factors. As McKenzie has suggested, with this shift ‘the goal is no longer supporting individual attempts to find happiness, rather [it is] experiencing the ups and downs of life within a meaningful social narrative’ [51]. Given this, we might ask, what would it mean to have ‘a meaningful social narrative’ for physicians to experience the ups and downs of their work?

A first step might be to normalize these ups and downs and openly discuss how we make sense of them, paying attention to the social, political and economic factors that shape this sense-making. Economics, for instance, is part of this social narrative, but absolute income is less important than relative income [50]. Attention to such factors might helpfully complicate discussions about the place of physician income in wellbeing at work and draw our attention to questions of the relative value of health professions and medical specialties in inter and intra professional workplaces. Other social factors that matter for a meaningful social narrative of the ups and downs of physician work include ‘a thriving and collaborative group identity’ [49] which this literature connects to the promise of constructive dissensus. To further explore this promise, scholarship might begin to connect the wellbeing conversation to the conversation about teamwork to understand how the ability to negotiate shared meaning in moments of conflict influences physician contentment.

### Individual happiness is not objective, it is subjective

The definition of happiness is multifaceted. Terminologies vary, and there remain no standardised definitions [29]. But what most terms have in common is that they approach happiness as an objective thing – something we can ‘get’ more or less of. However, our results suggest agreement that happiness is subjective: it is something we perceive and even something we manufacture thanks to our psychological immune system. Knowing that happiness is subjective, unstable, situated – even synthetic – calls into question our insistence on measuring it. Most of the records about physician happiness from medical education were measurement-focused. A critical examination of both our insatiable appetite to measure and the individual measures themselves is necessary if we are to avoid oversimplifying the construct of happiness as we integrate it more fully into medical education. Notwithstanding the question of whether happiness can be measured at all, our results suggest that understanding happiness may have more to do with measuring expectations and interpretations than experiencing happiness. Given this, we might explore the expectations and interpretations medical trainees bring to the profession, how they change over time, and how these changes influence happiness at work.

### Happiness is not straightforwardly good; it is also potentially coercive

Critical sociological approaches to happiness draw our attention to the role of happiness as ‘a central pacifying rhetoric and important technolog[y] for the management of subjectivity to ensure … the perpetuation of the existing order of things’ [29]. Such critiques of happiness remind us that, amid our efforts to promote the happiness of individual physicians, we must also be critically reflexive. We need to ask questions such as: Who defines ‘happiness’ for physicians? What social norms and power relations does that definition serve? Does our conceptualization of physician happiness acknowledge cultural contingencies? We are not suggesting that we should abandon initiatives that seek to improve happiness; however, as we pursue them, we must also ask ourselves how these perpetuate an existing structural and political order that is ‘coercive’, sustaining unacknowledged power relations. To use Ahmed’s term, do we cast as ‘killjoys’ [52] physicians who refuse to perpetuate the existing order by refusing to be happy-as-prescribed? Do we support physicians to be unhappy in the face of tragedy, or demand that they suppress that emotion and be happy (or at least neutral)? Recognizing that dominant understandings of happiness might disempower some physicians based on culture, gender, race or other minority factors, we should consider whether and how the prevalent conceptualization of happiness takes equality, diversity, inclusion and decolonisation (EDI-D) into account. As Slavin has recognized, ‘wellbeing programming has been a one-size-fits-all approach and has not adequately acknowledged and addressed the additional threats to wellbeing and satisfaction faced by many in our community’ [15]. To advance the conversation about physician happiness at work, we need to recognize how conventional definitions of physician happiness may be coercive, especially for minorities and equity deserving groups. We should be alert to physician unhappiness as a symptom not a disease – and even, perhaps, as a form of resistance to prescribed narratives.

### Limitations

A critical narrative review of happiness is, by design, selective and positioned rather than exhaustive and neutral [53]. We have chosen to highlight disciplines and concepts of happiness specifically related to our focus on physician happiness in the workplace. Consequently, our review examines the concept of workplace happiness as one important component of happiness in medical education: other aspects of happiness beyond ‘workplace happiness’ are likely relevant but are not captured in this work. We have focused on a subset of disciplines (psychology, sociology, economics, organizational behavior), and we acknowledge that our review of concepts of happiness in each discipline is selective rather than comprehensive: it reflects the records our search returned and there may be other insights about happiness in these disciplines that future work building on our own could explore. Furthermore, the inclusion of additional disciplines would probably enrich and expand the conceptualizations of happiness we have described in this work. A particular gap is the lack of philosophy records captured in our search, which initially surprised us. After consulting with a philosophy scholar, we determined that this was because the terminology in philosophy differs significantly from our search terms. Likely other disciplines, such as religious studies or history, were also outside the scope of our initial search due to differences in terminology for the happiness construct. This is a challenge for any review: reviewers need to decide how far from their original search terms to venture and where to draw the line. For this first narrative review of ‘happiness’ as it relates to physician happiness at work, we decided to remain consistent in our search terms and we therefore acknowledge that our results cannot be comprehensive of all treatments of the concept in all published literature. Finally, our review of included disciplines was not intended to be exhaustive, but to describe some key conceptualizations of happiness as they relate to understanding physician happiness at work. Undoubtedly there is much more to be learned about the history and nuances of each of these fields’ approaches to happiness.

## Conclusion

This critical narrative review has revealed that medical education rarely incorporates the concept of happiness in the workplace and, when it does, it draws exclusively from positive psychology. This disciplinary emphasis orients us to treat happiness as individual, objective, and necessarily good. Understanding organizational, economic and social aspects of happiness can usefully expand the conversation about physician wellbeing at work to include the insights that happiness is also social, subjective, and potentially coercive. With such insights, we might imagine different solutions to the persistent problem of physician wellbeing – in fact, we might even redefine the problem itself.

## References

[1] West CP, Dyrbye LN, Shanafelt TD. Physician burnout: contributors, consequences and solutions. J Int Med. 2018; 283(6): 516–29. DOI: 10.1111/joim.12752 29505159

[2] Shanafelt TD, Boone S, Tan L, Dyrbye LN, Sotile W, Satele D, et al. Burnout and satisfaction with work-life balance among US physicians relative to the general US population. Arch Intern Med. 2012; 172(18): 1377–85. DOI: 10.1001/archinternmed.2012.3199 22911330

[3] Yates SW. Physician Stress and Burnout. Am J Med. 2020; 133(2): 160–4. DOI: 10.1016/j.amjmed.2019.08.034 31520624

[4] Aggarwal R, Deutsch JK, Medina J, Kothari N. Resident Wellness: An Intervention to Decrease Burnout and Increase Resiliency and Happiness. MedEdPORTAL. 2017; 13: 10651. DOI: 10.15766/mep_2374-8265.10651 30800852 PMC6338253

[5] Panagioti M, Panagopoulou E, Bower P, Lewith G, Kontopantelis E, Chew-Graham C, et al. Controlled Interventions to Reduce Burnout in Physicians: A Systematic Review and Meta-analysis. JAMA Intern Med. 2017; 177(2): 195–205. DOI: 10.1001/jamainternmed.2016.7674 27918798

[6] Epstein RM. Mindful practice. JAMA. 1999; 282(9): 833–9. DOI: 10.1001/jama.282.9.833 10478689

[7] Eckleberry-Hunt J, Van Dyke A, Lick D, Tucciarone J. Changing the Conversation From Burnout to Wellness: Physician Well-being in Residency Training Programs. J Grad Med Educ. 2009; 1(2): 225–30. DOI: 10.4300/JGME-D-09-00026.1 21975983 PMC2931235

[8] Eckleberry-Hunt J, Kirkpatrick H, Taku K, Hunt R, Vasappa R. Relation Between Physicians’ Work Lives and Happiness. South Med J. 2016; 109(4): 207–12. DOI: 10.14423/SMJ.0000000000000437 27043800

[9] Fox S, Lydon S, Byrne D, Madden C, Connolly F, O’Connor P. A systematic review of interventions to foster physician resilience. Postgrad Med J. 2018; 94(1109): 162–70. DOI: 10.1136/postgradmedj-2017-135212 29018095

[10] Robertson HD, Elliott AM, Burton C, Iversen L, Murchie P, Porteous T, et al. Resilience of primary healthcare professionals: a systematic review. Br J Gen Pract. 2016; 66(647): e423–33. DOI: 10.3399/bjgp16X685261 27162208 PMC4871308

[11] Epstein RM, Krasner MS. Physician resilience: what it means, why it matters, and how to promote it. Acad Med. 2013; 88(3): 301–3. DOI: 10.1097/ACM.0b013e318280cff0 23442430

[12] Shanafelt TD, Noseworthy JH. Executive Leadership and Physician Well-being: Nine Organizational Strategies to Promote Engagement and Reduce Burnout. Mayo Clin Proc. 2017; 92(1): 129–46. DOI: 10.1016/j.mayocp.2016.10.004 27871627

[13] Shanafelt TD, Sloan JA, Habermann TM. The well-being of physicians. Am J Med. 2003; 114(6): 513–9. DOI: 10.1016/S0002-9343(03)00117-7 12727590

[14] Dyrbye LN, Thomas MR, Shanafelt TD. Medical student distress: causes, consequences, and proposed solutions. Mayo Clin Proc. 2005; 80(12): 1613–22. DOI: 10.4065/80.12.1613 16342655

[15] Slavin S. Reimagining Well-Being Initiatives in Medical Education: Shifting From Promoting Wellness to Increasing Satisfaction. Acad Med. 2021; 96(5): 632–4. DOI: 10.1097/ACM.0000000000004023 33635840

[16] Association of American Medical Colleges. Medical School Graduation Questionnaire 2019 All Schools Summary Report https://www.aamc.org/media/33566/download (accessed May 10, 2023).

[17] Association of American Medical Colleges. Medical School Year Two Questionnaire 2019 All Schools Summary Report https://www.aamc.org/media/43656/download (accessed May 10, 2023).

[18] Gerada C. Doctors and mental health. Oxford University Press UK. 2017; 660–1. DOI: 10.1093/occmed/kqx090 29301056

[19] Mata DA, Ramos MA, Bansal N, Khan R, Guille C, Di Angelantonio E, et al. Prevalence of depression and depressive symptoms among resident physicians: a systematic review and meta-analysis. JAMA. 2015; 314(22): 2373–83. DOI: 10.1001/jama.2015.15845 26647259 PMC4866499

[20] Raudenská J, Steinerová V, Javůrková A, Urits I, Kaye AD, Viswanath O, et al. Occupational burnout syndrome and post-traumatic stress among healthcare professionals during the novel coronavirus disease 2019 (COVID-19) pandemic. Best Pract Res Clin Anaesthesiol. 2020; 34(3): 553–60. DOI: 10.1016/j.bpa.2020.07.008 33004166 PMC7367798

[21] Shreffler J, Petrey J, Huecker M. The impact of COVID-19 on healthcare worker wellness: a scoping review. West J Emer Med. 2020; 21(5): 1059. DOI: 10.5811/westjem.2020.7.48684 PMC7514392 32970555

[22] Steptoe A. Happiness and health. Annu Rev Public Health. 2019; 40(1): 339–59. DOI: 10.1146/annurev-publhealth-040218-044150 30601719

[23] Stone AA, Mackie CE. Subjective well-being: measuring happiness, suffering, and other dimensions of experience. National Academies Press; 2013. 24432436

[24] Collier R. Physician health: beyond wellness to happiness. CMAJ. 2017; 189(39): E1242–3. DOI: 10.1503/cmaj.1095499 28970267 PMC5628042

[25] Baethge C, Goldbeck-Wood S, Mertens S. SANRA-a scale for the quality assessment of narrative review articles. Res Int Peer Rev. 2019; 4: 5. DOI: 10.1186/s41073-019-0064-8 PMC6434870 30962953

[26] Kahlke R, Lee M, Eva K. Critical Reviews in Health Professions Education Research. J Grad Med Educ. 2023; 15(2): 180–5. DOI: 10.4300/JGME-D-23-00154.1 37139200 PMC10150820

[27] Greenhalgh T, Thorne S, Malterud K. Time to challenge the spurious hierarchy of systematic over narrative reviews? Eur J Clin Invest. 2018; 48(6): e12931. DOI: 10.1111/eci.12931 29578574 PMC6001568

[28] Xu H, Remick DG. Pathology: A Satisfying Medical Profession. Acad Pathol. 2016; 3: 2374289516661559. DOI: 10.1177/2374289516661559 PMC5497859 28725775

[29] Frawley A. Happiness research: A review of critiques. Sociology Compass. 2015; 9(1): 62–77. DOI: 10.1111/soc4.12236

[30] Csikszentmihalyi, M. Flow: The psychology of optimal experience. New York: Harper & Row; 1990.

[31] Csikszentmihalyi, M. Flow, the secret to happiness https://www.ted.com/talks/mihaly_csikszentmihalyi_flow_the_secret_to_happiness (accessed July 06 2022).

[32] Csikszentmihalyi M, LeFevre J. Optimal experience in work and leisure. J Pers Soc Psychol. 1989; 56(5): 815. DOI: 10.1037/0022-3514.56.5.815 2724069

[33] McQueen S, Jiang S, McParland A, Hammond Mobilio M, Moulton CA. Cognitive flow in health care settings: A systematic review. Med Educ; 2020. DOI: 10.1111/medu.14435 33314200

[34] Tandler N, Krauss A, Proyer RT. Authentic happiness at work: Self-and peer-rated orientations to happiness, work satisfaction, and stress coping. Front Psychol. 2020; 1931. DOI: 10.3389/fpsyg.2020.01931 32849134 PMC7426460

[35] Singh S, Aggarwal Y. Happiness at Work Scale: Construction and Psychometric Validation of a Measure Using Mixed Method Approach. Journal of Happiness Studies. 2018; 19(5): 1439–63. DOI: 10.1007/s10902-017-9882-x

[36] Gilbert D. The surprising science of happiness [Internet]; 2004. Podcast.

[37] Gilbert D. Stumbling on happiness. New York, NY, US: Alfred A. Knopf; 2006. xviii, 277–xviii, p.

[38] Compton, WC, Hoffman, E. Positive psychology: The science of happiness and flourishing. Sage Publications; 2019.

[39] Ivtzan I, Lomas T. Mindfulness in positive psychology: The science of meditation and wellbeing. Routledge; 2016. DOI: 10.4324/9781315747217

[40] Seligman, ME. Flourish: A visionary new understanding of happiness and well-being. Simon and Schuster; 2012.

[41] Rehwaldt R, Kortsch T. Was macht bei der Arbeit glücklich? Zeitschrift für Arbeits- und Organisationspsychologie A&O. 2022; 66(2): 72–86. DOI: 10.1026/0932-4089/a000373

[42] Wright TA. Putting your best “face” forward: The role of emotion-based well-being in organizational research. Journal of Organizational Behavior. 2014; 35(8): 1153–68. DOI: 10.1002/job.1967

[43] Fisher CD. Happiness at Work. International Journal of Management Reviews. 2010; 12(4): 384–412. DOI: 10.1111/j.1468-2370.2009.00270.x

[44] Roy R. Workplace Happiness: A Conceptual Framework. 2020.

[45] Salas-Vallina A. Towards a Sustainable Leader-Follower Relationship: Constructive Dissensus, Organizational Virtuousness and Happiness at Work (HAW). Sustainability. 2020; 12(17): 7087. DOI: 10.3390/su12177087

[46] Sender G, Nobre GC, Armagan S, Fleck D. In search of the Holy Grail: a 20-year systematic review of the happy-productive worker thesis. International Journal of Organizational Analysis. 2021; 29(5): 1199–224. DOI: 10.1108/IJOA-09-2020-2401

[47] Kaul A, Sen C. Engaged Workforce: A Key Ingredient for Happiness at Work. Indian Journal of Psychological Science. 2015; 5(2): 42–5.

[48] Wahyanto T, Damayanti NA, Supriyanto S, Hartini S. Effect of Happiness at Work on Employee Engagement and Intention to Stay of Hospital Employees. Indian Journal of Public Health Research & Development. 2019; 10(12). DOI: 10.37506/v10/i12/2019/ijphrd/192170

[49] Davies W. The happiness industry: How the government and big business sold us well-being. Verso books; 2015.

[50] Binswanger M. Die Tretmühlen des Glücks: Herder; 2019.

[51] McKenzie J. Deconstructing happiness: critical sociology and the good life. New York: Routledge, Taylor & Francis Group; 2016. DOI: 10.4324/9781315735931

[52] Ahmed S. Killing joy: Feminism and the history of happiness. Signs: Journal of Women in Culture and Society. 2010; 35(3): 571–94. DOI: 10.1086/648513

[53] Greenhalgh T, Thorne S, Malterud K. Time to challenge the spurious hierarchy of systematic over narrative reviews? Eur J Clin Invest. 2018; 48(6). DOI: 10.1111/eci.12931 PMC6001568 29578574

